# Casticin inhibits epithelial-mesenchymal transition of liver cancer stem cells of the SMMC-7721 cell line through downregulating Twist

**DOI:** 10.3892/ol.2014.1899

**Published:** 2014-02-21

**Authors:** MENG HE, XIAO-CHENG CAO, GUI-CHENG HE, XI-FENG SHENG, XIAO-HONG AI, YOU-HUA WU

**Affiliations:** 1 Department of Oncology, The First Affiliated Hospital of University of South China, Hengyang, Hunan 421001, P.R. China; 2 Laboratory of Medicine Engineering, Medical College, Hunan Normal University, Changsha, Hunan 410013, P.R. China

**Keywords:** hepatocellular carcinoma, casticin, epithelial-mesenchymal transition, Twist

## Abstract

The existence of cancer stem cells (CSCs) is central to the pathogenesis and therapeutic target of human hepatocellular carcinoma. The aim of this study was to investigate the effects of casticin on epithelial-mesenchymal transition (EMT) of liver cancer stem cells (LCSCs) derived from the SMMC-7721 cell line. Our results demonstrated that CD133^+^ sphere-forming cells (SFCs) sorted from the SMMC-7721 cell line not only possessed a higher capacity to form tumor spheroids *in vitro*, but also had a greater potential to form tumors when implanted in Balb/c-nu mice, indicating that CD133^+^ SFCs possessed similar traits to LCSCs. Casticin increased the expression levels of E-cadherin and decreased those of N-cadherin in LCSCs. Treatment of LCSCs with casticin for 48 h also decreased the levels of the EMT-associated transcription factor, Twist. Overexpression of Twist attenuated the casticin-induced regulation of E-cadherin and N-cadherin protein expression, as well as the EMT capacity of LCSCs. In conclusion, CD133^+^ SFCs of the SMMC-7721 cell line may represent a subpopulation of LCSCs with the characteristics of EMT. Furthermore, casticin targeted LCSCs through the inhibition of EMT by downregulating Twist.

## Introduction

Hepatocellular carcinoma (HCC) is the third most common lethal malignancy and the fifth most common type of cancer worldwide. In 2008, there were an estimated 748,300 new liver cancer cases and 695,900 cancer mortalities worldwide (1,2). Half of these cancer cases and cancer-associated mortalities were estimated to occur in China (3). Liver transplantation or surgical resection remain the first-line treatments for HCC (4). However, patients often present symptoms at the advanced stages of HCC and, therefore, the first-line treatments are not effective for the majority of HCC cases. Due to high recurrence rates, even following surgical resection, the long-term prognosis of HCC remains unsatisfactory. For patients with HCC at the advanced stages, chemotherapy via either transarterial chemoembolization or a systemic route is the second-line treatment. Unfortunately, the efficacy of the regimens is limited due to the highly chemoresistant nature of the tumor and the toxicity of traditional chemotherapy (5–7).

The results of previous studies support the theory that tumors may be initiated and maintained by a small population of cells that have stem-like features, and this highly tumorigenic cell subpopulation within the tumor bulk has been considered as cancer stem cells (CSCs) (8). CD133^+^ liver CSCs were first reported to mark a CSC subpopulation in HCC by Suetsugu *et al* (9). Subsequently, Yin *et al* found that CD133^+^ cells isolated from HCC SMMC-7721 cells demonstrated an enhanced clonogenicity *in vitro* and tumorigenicity *in vivo* (10). Several studies on HCC cell lines have reported that CD133^+^ liver cancer stem cells (LCSCs) had an enhanced ability to differentiate and self-renew, were highly proliferative, demonstrated epithelial-mesenchymal transition (EMT), formed sphere clusters and had greater tumorigenicity and chemoresistance (11,12).

EMT is a crucial and dynamic process that facilitates the invasion of cancer cells. The induction of EMT was found to generate CSCs in breast cancer (13,14). Several studies have reported a conjunction between CSCs and EMT. EMT refers to morphological and molecular changes that occur when epithelial cells lose their characteristics and acquire mesenchymal properties, such as increased invasive and motility features (15). The expression of Twist, along with the expression of mesenchymal markers, such as N-cadherin, and the loss of E-cadherin, are key molecular markers of EMT (16). Transcription factors, including Twist and Snail, bind to consensus E-box sequences in the E-cadherin gene promoter and downregulate E-cadherin transcription. Twist, a transcription factor of the basic helix-loop-helix class that represses E-cadherin, has been reported to regulate HCC metastasis through the induction of EMT (17). The strategies aimed at targeting EMT of CSCs represent rational approaches for cancer prevention and treatment (18). This may prompt us to discover more preventive strategies for cancer management by reducing cancer resistance and recurrence, and improving patient survival. Several studies have reported that sulforaphane has the capability to suppress pancreatic tumor initiating cells and breast CSCs (19,20). These studies provide a basis for preclinical and clinical evaluation of dietary compounds for chemoprevention of CSCs.

Casticin is one of the main active components from Fructus Viticis (Chinese name, Manjingzi), which has been shown to inhibit the proliferation of breast cancer (21), lung cancer (22), colon cancer (23) and human myelogenous leukemia cells (24) *in vitro,* as well as exerting an anti-inflammatory effect *in vivo* (25). Casticin was also reported to induce apoptosis (26), growth suppression and cell cycle arrest through the activation of FOXO3a in HCC cell lines (27). However, to the best of our knowledge, to date there are no studies regarding the inhibition of casticin on LCSCs.

In the present study, we aimed to investigate the effects of casticin on the EMT of LCSCs derived from the SMMC-7721 cell line.

## Materials and methods

### Cell culture and reagents

The human HCC SMMC-7721 cell line was obtained from the Chinese Academy of Sciences (Shanghai, China). Cells were maintained in Dulbecco’s modified Eagle’s medium (DMEM) supplemented with 10% fetal bovine serum (FBS; Hangzhou Sijiqing Biological Engineering Materials Co., Ltd., Hangzhou, China), 100 U/ml penicillin and 100 μg/ml streptomycin (Invitrogen Life Technologies, Carlsbad, CA, USA) in an incubator containing 5% CO_2_ at 37°C. Casticin was purchased from Chengdu Biopurify Phytochemicals Ltd. (purity, ≥98%; Chengdu, China) and dissolved in dimethyl sulfoxide (DMSO) as a 10 mmol/l stock solution, and diluted in a medium to the indicated concentration before use. Trypsin and DMSO were purchased from Amersco Inc. (Solon, OH, USA).

### Cell sorting and sphere culture

Cells were labeled with primary CD133/1 antibody [mouse immunoglobulin 1 (IgG1), 1 μl per million cells; Miltenyi Biotec, Bergisch Gladbach, Germany], subsequently magnetically labeled with rat anti-mouse IgG1 microbeads (20 μl per 10 million cells; Miltenyi Biotec) and separated on MACS LS column (Miltenyi Biotec). All procedures were performed according to the manufacturer’s instructions. The purity of sorted cells was evaluated by flow cytometry (FCM) and western blot analysis. The FCM was performed with Epics Altra Cell Sorter (Beckman Coulter, Inc., Pasadena, CA, USA) using CD133/1 primary antibody (Miltenyi Biotec) and phycoerythrin-conjugated secondary antibody (Sigma-Aldrich, St. Louis, MO, USA). The CD133^+^ cells and the parental cells were collected and washed to remove serum, and then suspended in serum-free DMEM/F12 supplemented with 20 ng/ml human recombinant epidermal growth factor (Invitrogen Life Technologies), 20 ng/ml human recombinant basic fibroblast growth factor (Invitrogen Life Technologies), 2% B27 supplement without vitamin A (Invitrogen Life Technologies), 0.4% bull serum albumin (Invitrogen Life Technologies), 4 ng/ml insulin (Invitrogen Life Technologies), 100 IU/ml penicillin and 100 μg/ml streptomycin. The cells were subsequently cultured in ultra low attachment six-well plates (Corning Inc., Corning, NY, USA) at a density of no more than 2,000 cells/well.

### Spheroid passage and sphere formation assay

The CD133^+^ sphere-forming cells (SFCs) of the SMMC-7721 cell line were collected by gentle centrifugation, then dissociated with trypsin-EDTA and mechanically disrupted with a pipette. The resulting single cells were centrifuged to remove the enzyme, resuspended in stem cell-conditioned culture medium and allowed to reform spheres. The tumor spheres were passaged every 6 days before they reached a diameter of 50 mm. The volume of HCC spheres was calculated by the following: V = (4/3)πr^3^, where r is the radius of the sphere. The number and volume of the spheres was observed and compared. To examine the effects of casticin on the sphere formation, the resulting single cells with a density of 2×10^3^ cells/ml were plated to ultra low attachment six-well plates and supplemented with serum-free medium containing various concentrations of casticin.

### In vivo tumorigenicity assay

Pathogen-free male Balb/c-nu mice (age, 4–5 weeks) were purchased from the Animal Institute of the Chinese Academy of Medical Science (Beijing, China). All animal studies were performed in accordance with the standard protocols approved by the ethics committee of the Hunan Normal University (Changsha, China) and the committee of experimental animal feeding and management. Male Balb/c-nu mice (age, 4–5 weeks) were randomly divided into five groups (four mice per group) and maintained under standard conditions, according to the standard protocols. Cells were suspended in serum free-DMEM/Matrigel (BD Biosciences, Franklin Lakes, NJ, USA) mixture (1:1 volume). Each Balb/c-nu mouse was inoculated subcutaneously with different numbers of CD133^+^ SFCs (5×10^2^, 1×10^3^, 5×10^3^, 1×10^4^ and 5×10^4^ cells) in one flank and parental cells (5×10^4^, 1×10^5^, 2×10^5^, 5×10^5^ and 1×10^6^ cells) in the other, respectively. Tumorigenicity experiments were terminated 2 months after cell inoculation. Tumor size was measured with a caliper and the volume was calculated using the following: V (mm^3^) = L×W^2^×0.5; where L is the length and W is the width. Harvested tumors were examined and weighed immediately. Moreover, specimens from tumor tissue samples were fixed in 10% neutral-buffered formalin, processed in paraffin blocks and sectioned. The tissue sections were stained with hematoxylin and eosin and examined under light microscopy (IX-70, Olympus Corporation, Tokyo, Japan) for identification of the histopathological changes.

### pcDNA3-Twist1 transfection

pcDNA3-Twist1 was purchased from GenePharma (Shanghai, China). To generate Twist1-expressing stable transfectants, the parental cells and SFCs of the SMMC-7721 cell line were transfected with pcDNA3-Twist1, and stable clones were selected with 1,000 μg/ml of G418 (EMD Millipore, San Diego, CA, USA) for 4 weeks.

### Western blot analysis

Protein extracts were resolved by 12% SDS-PAGE and transferred onto nitrocellulose membranes (Amersham Bioscience, Shanghai, China) blocked in 5% skimmed milk. Primary antibodies were used as indicated by the manufacturer’s instructions and are as follows: Mouse anti-CD133 (Santa Cruz Biotechnology, Inc., Santa Cruz, CA, USA), Twist (Cell Signaling Technology, Inc., Beverly, MA, USA), N-cadherin (EMD-Millipore) and E-cadherin (BD Biosciences). After incubation with horseradish peroxidase-conjugated anti-mouse or anti-rabbit secondary antibodies (Amersham Biosciences, Cardiff, UK), specific protein bands were visualized by enhanced chemiluminescence (Amersham Biosciences). β-actin (Sigma-Aldrich) was used as a loading control.

### Statistical analysis

All values in the figures and text are expressed as the means ± SD. Statistical analyses were performed using SPSS software, version 16.0 (SPSS, Inc., Chicago, IL, USA). Any significant differences among the mean values were evaluated by the Student’s t-test. A two-sided P<0.05 was considered to indicate a statistically significant difference.

## Results

### Sorting and identification of LCSCs derived from the HCC SMMC-7721 cell line

**Figure 1:**
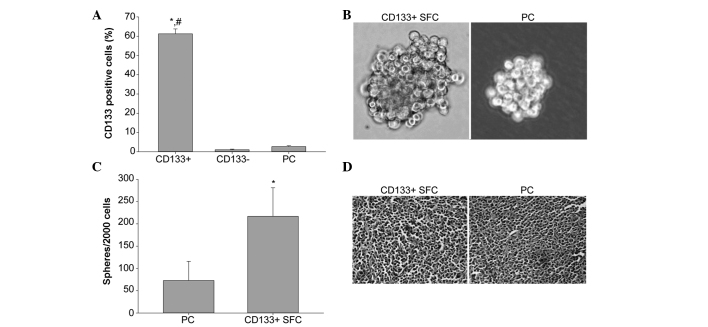
CD133^+^ SFCs derived from the SMMC-7721 cell line enriched liver cancer stem cells. (A) CD133^+^ cell subpopulation sorted from the hepatocellular carcinoma SMMC-7721 cell line by MACS overexpressed the stem cell surface marker, CD133, detected by flow cytometry using phycoerythrin-conjugated antihuman CD133 antibody, respectively. Data are expressed as the means ± SD (n=3). ^*^P<0.05 significant difference from the CD133^+^ cells. ^#^P<0.05 significant difference from the parental cells. (B) CD133^+^ cells from the SMMC-7721 cell line and the parental cells formed liver cancer spheroids in stem cell-conditioned medium (magnification, ×100). (C) CD133^+^ cells from the SMMC-7721 cell line and the parental cells formed liver cancer spheroids in stem cell-conditioned medium. Data are expressed as the means ± SD (n=3). ^*^P<0.05 significant difference from the parental cells. (D) Hematoxylin and eosin staining revealed similar histological characteristics in tumor xenografts derived from CD133^+^ SFCs of the SMMC-7721 cell line and the parental cells (magnification, ×100). SFCs, sphere-forming cells.

In order to isolate LCSCs, SMMC-7721 cells were enzymatically dispersed into single-cell suspensions and incubated using an anti-CD133 antibody (Miltenyi Biotec) and then analyzed by FCM. Following separation on MACS LS column for purity, FCM analysis showed that the CD133-positive cell percentage of CD133^+^ subpopulation was 61.24±2.54%, which was obviously higher than that of CD133^−^ population (1.07±0.34%) and the parental cells (2.72±0.46%), indicating our immunomagnetic separation system was efficient (Fig. 1A).

We performed the tumor sphere formation assay with stem cell-conditioned medium to enrich and expand LCSCs from sorted CD133^+^ cells of the SMMC-7721 cell line. In the case of inoculation of 2,000 cells per well, an increased number of spheres formed in the group of CD133^+^ cells, while at the same time as sphere formation, the sphere volume of CD133^+^ cells was greater than that of the parental cells (Fig. 1B and C).

To examine the tumor initiating capability, the male Balb/c-nu mice were transplanted with various amounts of CD133^+^ SFCs of the SMMC-7721 cell line and parental cells. Our results demonstrated that as few as 1,000 CD133^+^ SFCs were sufficient to induce tumor development, whereas, at least 2×10^5^ SMMC-7721 parental cells were necessary to consistently generate a tumor in the same model and required a longer time period (Table I). Hematoxylin and eosin staining indicated that CD133^+^ SFCs formed similar histological features as the tumor xenografts in the parental cells (Fig. 1D). These results demonstrated that CD133^+^ SFCs sorted from the SMMC-7721 cell line were highly tumorigenic and possessed LCSC properties.

### Casticin inhibits EMT of LCSCs derived from the HCC SMMC-7721 cell line

**Figure 2:**
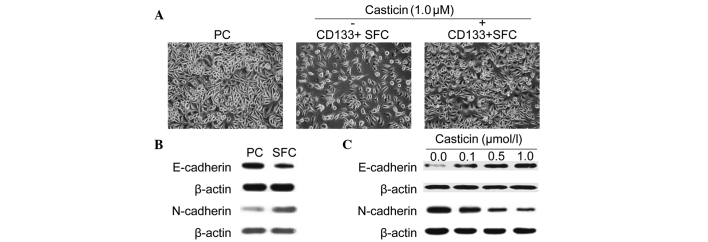
Casticin inhibited EMT of liver cancer stem cells derived from the SMMC-7721 cell line. (A) Casticin inhibited EMT. The CD133^+^ SFCs of the SMMC-7721 cell line were grown in plates and treated with 1.0 μmol/l casticin. Cell morphology tended to transform to the epithelial phenotype. (B) CD133^+^ SFCs expressed a higher N-cadherin protein levels, which was typically associated with mesenchymal cells, and a lower expression of epithelium-associated E-cadherin protein. (C) Treatment of casticin resulted in downregulation of N-cadherin and the upregulation of E-cadherin in CD133^+^ SFCs of the SMMC-7721 cell line. EMT, epithelial-mesenchymal transition; SFCs, sphere-forming cells.

EMT is a key process during the metastasis of LCSCs. Therefore, we sought to examine whether morphological changes existed between LCSCs and parental SMMC-7721 cells cultured adherently *in vitro*. As shown in Fig. 2A, LCSCs exhibited a spindle-like shape, while parental cells displayed a cobble-stone-like phenotype. However, treatment with 1.0 μmol/l casticin suppressed EMT in LCSCs as morphological changes from a spindle-like shape to a cobble-stone-like appearance were observed.

Moreover, similar results were further confirmed by western blotting using specific antibodies against EMT-relative markers. As shown in Fig. 2B, CD133^+^ SFCs expressed a higher N-cadherin protein level, which is typically associated with mesenchymal cells and a lower expression of epithelium-associated E-cadherin protein. Casticin, at increasing concentrations, induced upregulation of the epithelial marker, E-cadherin, and downregulation of the mesenchymal marker, N-cadherin, after treatment for 24 h in CD133^+^ SFCs derived from the SMMC-7721 cell line (Fig. 2C). These results demonstrated that casticin possessed inhibitory effects of EMT in LCSCs purified from the SMMC-7721 cell line.

### Casticin downregulates the expression of Twist in LCSCs derived from the HCC SMMC-7721 cell line

**Figure 3:**
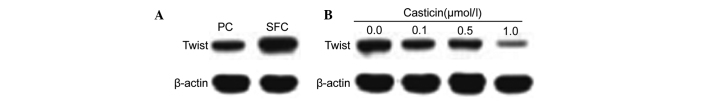
Casticin inhibited the expression of Twist in liver cancer stem cells derived from the hepatocellular carcinoma SMMC-7721 cell line. (A) Western blot analysis showed that Twist was highly expressed in CD133^+^ SFCs of the SMMC-7721 cell line. (B) Casticin downregulated the expression of Twist protein in a dose-dependent manner. SFCs, sphere-forming cells.

The transcription factor, Twist, was identified as the critical molecule of EMT (27,28). Based on our results that casticin inhibited EMT in LCSCs, we further investigated the effects of casticin on the expression of Twist. Twist was highly expressed in CD133^+^ SFCs (Fig. 3A). Western blot analysis indicated that the protein levels were downregulated following treatment with casticin (0.0, 0.1, 0.5 and 1.0 μmol/l) in CD133^+^ SFCs (Fig. 3B).

### Overexpression of Twist reduced the inhibition of EMT in LCSCs and self-renewal by casticin

**Figure 4:**
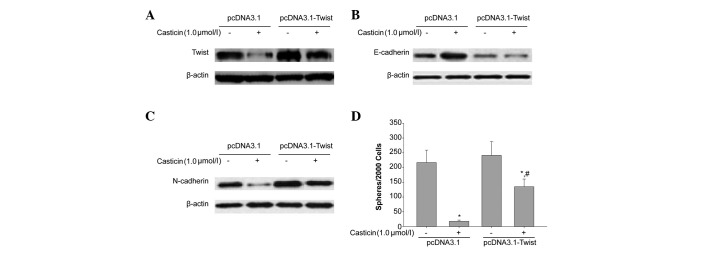
Overexpression of Twist attenuated the inhibitory effects of casticin on the epithelial-mesenchymal transition in LCSCs and self-renewal of LCSCs derived from the SMMC-7721 cell line. (A) Western blot analysis showed that the overexpression of Twist in CD133^+^ SFCs displayed a deregulated response to casticin. (B) Western blot analysis showed lower expression of E-cadherin after transfection with plasmid pcDNA3.1-Twist in SFCs of the SMMC-7721 cell line. Overexpression of Twist reduced the effects of casticin-induced upregulation of E-cadherin. (C) Western blot analysis showed overexpression of N-cadherin after transfection with plasmid pcDNA3.1-Twist in SFCs of the SMMC-7721 cell line. Overexpression of Twist reduced the effects of casticin-induced downregulation of N-cadherin. (D) The sphere forming ability of CD133^+^ SFCs derived from the SMMC-7721 cell line treated with casticin was partly rescued after overexpression of Twist. Data are expressed as the means ± SD, (n=4 per group). ^*^P<0.05, significantly different from 0.1% dimethyl sulfoxide-treated cells. ^#^P<0.05 significantly different from the cells treated with the same concentration of casticin using pcDNA3.1 transfection. LCSCs, liver cancer stem cells; SFCs, sphere-forming cells.

Casticin inhibited EMT of CD133^+^ SFCs, while the overexpression of Twist prevented casticin-induced inhibition of EMT. We transfected the plasmid, pCDNA3.1-Twist, and the negative control, pCDNA3.1, into CD133^+^ SFCs derived from SMMC-7721 cells. As shown in Fig. 4A, compared with the negative control vector, pCDNA3.1, CD133^+^ SFCs transfected with pCDNA3.1-Twist significantly overexpressed Twist. Furthermore, as shown in Fig. 4B and C, the overexpression of Twist attenuated casticin-induced upregulation of E-cadherin and downregulation of N-cadherin, respectively. This tumor sphere-forming experiment showed that the ectopic expression of Twist in this cell line promoted tumor sphere formation. We also treated CD133^+^ SFCs of SMMC-7721 cells transfected with pCDNA3.1-Twist with 1.0 μmol/l casticin. As shown in Fig. 4D, after the addition of casticin, the sphere-forming ability of CD133^+^ SFCs in this subpopulation was greater than that of the control group. Taken together, the overexpression of Twist partly reduced the inhibitory effect of casticin on EMT of CD133^+^ SFCs. These results further supported that the downregulation of Twist expression may contribute to the inhibitory effects of casticin on EMT of LCSCs derived from the SMMC-7721 cell line.

## Discussion

Targeting LCSCs through repression of the EMT process is an emerging strategy for the treatment and prevention of HCC. As these interventions focus on EMT rather than toxicity induction, they are potentially less toxic than conventional chemotherapeutic agents. Our study confirmed that CD133^+^ SFCs sorted by MACS LS column from the SMMC-7721 human liver cancer cell line showed an enhanced capacity of EMT and had greater tumorigenicity than the parental cells, which indicated that CD133^+^ SFCs enriched LCSCs. Suetsugu *et al* and Yin *et al* demonstrated that CD133^+^ HCC cells were cancer stem/progenitor cells (9,10), which was consistent with our findings. Thus, CD133 could be regarded as a potential target of stem cell-targeted therapy for HCC.

EMT is a physiological phenotypic shift that facilitates invasion and metastasis of liver cancer cells. Motile and invasive tumor cells have been reported to gain the phenotypic and molecular characteristics of EMT, including the gene expression of EMT regulators, such as Twist (30). The ‘mobile CSCs’ were reported to derive from stationary CSCs in colon cancers that underwent a transient EMT (30), and the induction of an EMT by ectopic expression of Twist transcription factors has been reported to generate CSC properties in human breast cancer cells (13,14). Our data showed that the protein levels of the EMT regulator, Twist, were higher in CD133^+^ SFCs than that in the parental cells. These results were consistent with previous reports that Twist was involved in infiltrative subtypes of HCC and breast cancer (31). This data may aid in explaining previous reports in which HCCs expressing CD133 had a poor survival and high recurrence (32).

E-cadherin, the best-characterized member of epithelial markers, was lost upon EMT, resulting in migration (33). Downregulation of E-cadherin and upregulation of N-cadherin have been reported in various tumors during EMT (34). In the SMMC-7721 cell line, CD133^+^ SFCs showed upregulated expression of N-cadherin and downregulated expression of E-cadherin compared with that of the parental cells, which suggested that EMT can more commonly occur in CD133^+^ SFCs. Thus, finding a natural agent that targets LCSCs and EMT is considered an emerging strategy for cancer prevention and recurrence.

In this study, cell morphology of CD133^+^ SFCs after treatment with casticin (1.0 μmol/l) was assessed. Consistent with our hypothesis, the CD133^+^ SFCs showed typical morphologic phenotypes of EMT, including loss of cell-cell adhesion, spindle-shaped morphology and increased formation of pseudopodia. However, following treatment with casticin (1.0 μmol/l) for 48 h, all these features disappeared and were displaced by epithelial cobble-stone-like cells. This phenomenon suggested that casticin can reverse the EMT process of CD133^+^ SFCs, morphologically. For further research, we assessed the expression of invasion- and EMT-associated proteins, E-cadherin and N-cadherin, of CD133^+^ SFCs after treatment with casticin at various concentrations. Notably, the expression of E-cadherin increased in a concentration-dependent manner in CD133^+^ SFCs, while N-cadherin decreased, indicating that casticin can reverse the EMT process by upregulating E-cadherin and downregulating N-cadherin. In addition, we also evaluated the EMT regulator, Twist, using western blot analysis after treatment with casticin at various concentrations. The results were consistent with our hypothesis; casticin downregulated the expression of the transcriptional factor, Twist. We also found that CD133^+^ SFCs transfected with the pCDNA3.1-Twist plasmid significantly overexpressed Twist compared with cells with the negative control vector (pCDNA3.1). Overexpression of Twist attenuated casticin-induced upregulation of E-cadherin and the downregulation of N-cadherin. In addition, the suppressive effects of casticin on EMT of CD133^+^ SFCs were partially rescued by the overexpression of Twist. These data demonstrated that casticin could inhibit the EMT of CD133^+^ SFCs by downregulating Twist. Considering the relatively non-toxic nature of casticin, targeting EMT-type cells and LCSCs by combining it with conventional chemotherapeutics may be a novel and safe approach for achieving an improved treatment outcome. However, further in-depth preclinical and clinical studies are warranted in order to appreciate its value in reversal of the EMT process on LCSCs.

In conclusion, our study demonstrated that casticin has the potential to target CD133^+^ SFCs of the SMMC-7721 cell line, namely LCSCs, and can inhibit the EMT process. Therefore, we suggest that casticin may be an effective candidate for the prevention and treatment of recurrence and metastasis as a cancer therapeutic agent for HCC.

## Figures and Tables

**Table I:** Tumorigenicity experiments of CD133^+^ SFCs and the parental cells in Balb/c-nu mice.

Cell type	Cell nos. injected	Tumor incidence^a^	Latency (days)^b^
SMMC-7721 parental cells	5×10^4^	0/4	-
	1×10^5^	0/4	-
	2×10^5^	3/4	41
	5×10^5^	4/4	35
	1×10^6^	4/4	12
CD133^+^ cells	5×10^2^	0/4	-
	1×10^3^	4/4	39
	5×10^3^	4/4	23
	1×10^4^	4/4	9
	5×10^4^	4/4	6

a The number of tumors detected/number of injections.

b Approximate number of days from tumor cell injection to appearance of a tumor.
